# Utility of muscle ultrasound in nutritional assessment of children with nephrotic syndrome

**DOI:** 10.1007/s00467-022-05776-y

**Published:** 2022-11-10

**Authors:** Mona Hamed Gehad, Yousif Mohamed Yousif, Maha Ibrahim Metwally, Amany Mohammed AbdAllah, Lamiaa Lotfy Elhawy, Amal S. El-Shal, Ghada Mohammed Abdellatif

**Affiliations:** 1 Faculty of Medicine, Department of Pediatrics, Zagazig University, Zagazig, Sharkia Governorate Egypt; 2 Faculty of Medicine, Radiodiagnosis Department, Zagazig University, Zagazig, Egypt; 3 Faculty of Medicine, Family Medicine Department, Zagazig University, Zagazig, Egypt; 4 Faculty of Medicine, Public Health and Community Medicine, Zagazig University, Zagazig, Egypt; 5 Faculty of Medicine, Medical Biochemistry Department, Zagazig University, Zagazig, Egypt

**Keywords:** Nephrotic, Malnutrition, Ultrasound, Protein energy wasting, Muscle wasting, Quadriceps muscle

## Abstract

**Background:**

Nutritional status assessment in children with nephrotic syndrome (NS) is critical for identifying patients who are at risk of protein-energy wasting (PEW) and for determining their nutritional needs and monitoring nutritional intervention outcomes.

**Methods:**

In a case–control study, we enrolled 40 children (age range: 2–16 years) with NS and 40 apparently healthy children (age and sex-matched) as a control group. Anthropometric data, as well as demographic, clinical, and laboratory data, were collected. A dietary intake assessment using a 3-day food intake record was done, and the quadriceps rectus femoris thickness (QRFT) and quadriceps vastus intermedius thickness (QVIT) were assessed using B-mode ultrasound and compared between both groups.

**Results:**

Children with NS had lower QRFT and QVIT measurements than control groups (*p* < 0.001). Inadequacy in protein intake occurred in 62.5% and 27.5% of the NS and control groups, respectively (*p* = 0.002). The thickness of the rectus and vastus muscles by ultrasound was significantly associated with the percentage of protein intake (*p* < 0.001). The ROC curve revealed that the best cutoff value of QRFT for the prediction of the patient at risk of malnutrition was ≤ 1.195 with an area under curve of 0.907, with *p* < 0.001.

**Conclusion:**

In children with NS, skeletal muscle ultrasound is a simple and easy-to-use bedside technique for the identification of patients at risk of malnutrition.

**Graphical abstract:**

**Figure:**
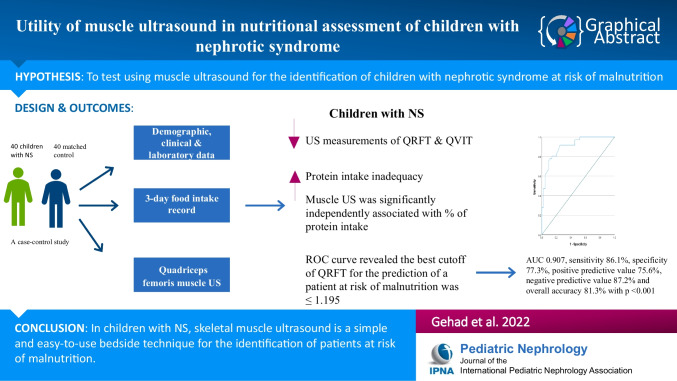
A higher resolution version of the Graphical abstract is available as Supplementary information

**Supplementary Information:**

The online version contains supplementary material available at 10.1007/s00467-022-05776-y.

## Introduction

Nephrotic syndrome (NS) is a common kidney disorder in children that causes proteinuria, hypoalbuminemia, and edema [1]. In healthy children, the annual incidence of NS is estimated to be two to seven new cases per 100,000 under the age of 18 [2].

Malnutrition is more common in children with NS because they are prone to both macro and micronutrient deficiencies, putting them at risk for stunted growth, muscle loss, and cognitive impairment. The disease process, poor dietary intake, and steroid therapy all contribute to nutritional impairment [3]. Nutrition is a modifiable risk factor for mortality in children with kidney abnormalities, and it should be checked on a regular basis.

The expert panel of the International Society of Renal Nutrition and Metabolism (ISRNM) has outlined four diagnostic criteria: biochemical criteria, low body weight, lower muscle mass, and lower protein and calorie intake. When three of the four are met, a diagnosis of protein-energy wasting (PEW) is made [4]. Proteinuria, in addition to the inflammation associated with chronic disease, can contribute to PEW, resulting in an accelerated loss of lean body mass (LBM), which can also have a negative impact on morbidity and mortality [5]. Protein-energy wasting associated with NS (neph-PEW) defines a specific metabolic pattern in patients with NS with elevated phosphorus, uric acid, blood urea, and lean tissue deficiency [6].

Pathophysiological studies have linked NS to a hypercatabolic state characterized by accelerated muscle mass loss as the increased hepatic synthesis is not enough to compensate for the total loss, resulting in a decreased rate of protein turnover of other protein pools and amino acid mobilization from muscle [6, 7].

The stimulatory effects of food and physical activity are critical in maintaining muscle mass. Amino acids and contractile activity are key stimuli for muscle protein synthesis, which is the main controlled variable regulating muscle mass [8]. Long-term steroid treatment in children with NS is known to increase risk of obesity, suppress protein synthesis, and have a detrimental impact on quality of life as well as augment the degradation of myofibrillar proteins [9]. Glucocorticoid-induced skeletal muscle atrophy causes fatigue in daily physical activities as well as a reduction in body movement, resulting in a decreased ability to perform physical activity [9]. Physical inactivity alters body composition and has been linked with increased inflammation and anabolic resistance [10].

The overall assessment of nutritional status is generally grounded on the patient’s history, physical assessment, evaluation of dietary intake, biochemical markers, and screening methods that provide a comprehensive representation of a patient’s protein and energy reserves, in addition to their nutritional risk with the goal of early detection and monitoring in patients at risk of muscle loss [11].

The current gold standard for clinical evaluation of nutritional intake in children with chronic kidney disease (CKD) is the 3-day diet history [12]. However, using it places an undue burden on responders, necessitating strong motivation, literacy, and the ability to analyze typical intake across several days. It also necessitates the participation of qualified nutritionists [13]. Moreover, food records are prone to systematic inaccuracies such as underreporting, as well as the tendency to influence and change eating habits during data collection, reducing the accuracy of consumption estimates [14].

Low muscle mass is one of the diagnostic requirements for malnutrition according to recent definitions [15, 16]. Several methodologies are available for the estimation of muscle mass for early detection and monitoring in patients at risk of muscle loss. Some of these techniques, such as bio-impedance analysis (BIA) and bio-impedance spectroscopy (BIS), allow for the assessment of muscle mass but are hampered by overhydration [17]. Other imaging-based methods (dual-energy X-ray absorptiometry (DEXA), magnetic resonance imaging (MRI), and computed tomography (CT)) are considered the “gold standard” techniques for evaluation of muscle mass, but their use is primarily limited to research purposes due to their high expense, inaccessibility at the bedside, exposure to radiation, and powerful magnetic fields [18].

More recently, muscle ultrasound (US) has become a popular method for bedside body composition analysis due to its portability, low price, easy accessibility, minimal training, and ease of use [19]. Data from these devices would lessen subjectivity in nutritionists’ assessments of muscularity, allowing for more sensitive screening for those at nutritional risk, better monitoring, and evaluation of nutrition interventions in hospitalized patients.

US measurements of quadriceps muscle, the single biggest skeletal muscle group in the body, are strongly correlated to muscle mass as determined by gold standard techniques. Particularly, DEXA data show that quadriceps femoris muscle thickness best correlates with fat-free mass [20] and seems to be as accurate as the estimation of muscle mass by CT or MRI [21]. Recently, it has been proposed that quadriceps femoris US is a valid and simple technique to measure muscle thickness in patients with acute kidney injury (AKI) and kidney failure [22, 23]. The same study found that even significant and fast fluid changes generated by kidney replacement treatment (KRT) in overhydrated patients had no effect on US quadriceps muscle thickness assessments [22].

There are currently no data on the use of the quadriceps femoris muscle US in children with NS. In the current study, we aimed to evaluate the utility of quadriceps femoris muscle US for the identification of children with NS at risk of malnutrition.

## Patients and methods

### Research design and patients

A case–control study was carried out between the periods of April 2021 and March 2022 at the nephrology unit of the pediatric department of Zagazig University Hospitals. The study included 40 children with NS aged 2 to 16 years who were on prednisone therapy and were initially diagnosed using clinical and laboratory data (generalized edema, serum protein/creatinine ratio > 2.0, dipstick urine protein, and serum albumin < 2.5 g/dL). The children were selected by a simple random method from our patient list. The control group consisted of 40 children who were apparently healthy children and presented to the outpatient general pediatric clinic for minor illnesses and a physical examination without proteinuria or overhydration. Children with co-existent comorbidities such as severe cardiovascular, respiratory, or hepatic disease, or cancer, were excluded from the study.

All children underwent full history-taking, and anthropometric measurements including dry weight and length were used for the calculation of the body mass index (BMI) as kg/m^2^.

### Biochemical measurements

Blood serum cholesterol, serum albumin, total protein, prealbumin, serum creatinine, uric acid (UA), and phosphorus (Pi) were tested using the appropriate chemical analyzer. Estimated glomerular filtration rate (eGFR) was calculated based on the Schwartz formula.

### Nutritional status assessment

A prospective 3-day food intake record was documented, which required the patient or family to keep track of everything the patient ate and drank in real time. Food records were analyzed using MyPlate Tracker from the U.S. Department of Agriculture, with a particular emphasis on the distribution of daily energy and protein intake in the children’s diets.

Food record analyses helped to compare the daily energy intake to recommended intake based on the calculations of estimated energy requirements (EERs) using age, sex, weight, and height (50th percentile height for weight) as well as a sedentary physical activity coefficient to estimate total energy needs while on steroids [24].

Percent contributions to total energy intake from protein were compared to the Acceptable Macronutrient Distribution Range (AMDR) for protein. According to the Food and Nutrition Board, Institute of Medicine, AMDR is defined as “a range of intakes for a particular energy source that is associated with reduced risk of chronic disease while providing adequate intakes of essential nutrients.” AMDR for protein range was based on 5–20% and 10–30% of energy intake for children aged 1 to 3 years and 4 to 18 years, respectively [24–27]. Patients with protein intakes lower than these recommended ranges are identified as being at risk of malnutrition.

### US measurements

The thicknesses of the quadriceps rectus femoris (QRF) and vastus intermedius (QVI) were evaluated using B-mode ultrasound (Philips HD7xe) with a high-frequency linear array transducer (7.5 MHz). All cases were examined regarding the right QRF and QVI muscle thicknesses. The patient was lying supine with extended, relaxed knees, and toes directed upward. Maximum thickness of each QVI and QRF muscle was assessed from the femur to the muscle’s inner edge and from the subcutaneous layer to the QRF muscle’s inner border, respectively. Additionally, we kept a standard level where the muscle thickness was measured, at the level of two particular landmarks, the midpoint, and the border between the upper two-thirds and the lower third between the upper pole of the patella and the superior anterior iliac spine [22]. For each individual muscle, multiple measurements were taken, and a mean was calculated and used for further analysis. Special precautions were taken in all cases, including placing the transducer perpendicular to the long axis of the thigh in a fixed orientation and using enough contact gel to guard the muscle from excessive pressure and compression.

### Statistical analysis

Statistical Package for Social Sciences was used to conduct the statistical analysis (SPSS version 20.0). According to the type of data, qualitative data is represented by numbers and percentages, while quantitative data is represented by mean and standard deviation. The following tests were used to determine the significance of differences: *t*-test for comparisons between parametric quantitative independent groups, Mann–Whitney in non-parametric, paired with paired *t*-test. The significance level was chosen at < 0.05 for significant results and < 0.001 for highly significant results.

## Results

### Clinical and demographic variables

The study included 40 children with NS and 40 participants within the control group. On comparing demographic data between both groups, there was a statistically non-significant difference between them concerning weight or height, though BMI was significantly higher among the NS group (median 18.85 kg/m^2^) compared to the control group (16.34 kg/m^2^). Disease duration ranged from 1 month to 14 years, with a median of 1 year (Table 1).

**Table 1:** Demographic and clinical data comparisons between study groups

	Group		Test	
	NS group	Control group	Test	*p*
	*N* = 40 (%)	*N* = 40 (%)		
Gender:				
Male	20 (50%)	24 (60%)	*χ*^2^ = 0.808	0.369
Female	20 (50%)	16 (40%)		
Age (year):				
Median (range)	6.75 (2–15)	5.5 (2–16)	*Z* = − 1.427	0.154
Weight (kg):				
Median (range)	22.75 (10.5–60)	19 (12–60)	*Z* = − 1.547	0.122
Height (m):				
Mean ± *SD*	1.16 ± 0.32	1.62 ± 0.2	*t* = 0.889	0.372
BMI (kg/m^2^):				
Mean ± *SD*	18.85 ± 4.98	16.34 ± 1.97	*t* = 2.966	0.005*
Duration (year)				
Median (range)	1 (0.08–14)			

*χ*^*2*^, chi-square test; *t*, independent sample *t*-test; *Z*, Mann–Whitney test

*p < 0.05 is statistically significant

### Biochemical measurements

The NS group had significantly lower serum total protein (mean 4.08 g/dL vs. 6.63 g/dL in the control group), albumin (mean 2.35 g/dL vs. 3.72 g/dL in the control group), and prealbumin (mean 9.18 g/dL vs. 18.39 g/dL in the control group). On the other hand, the NS group had significantly higher total cholesterol (mean 385.58 mg/dL vs. 140.25 mg/dL in the control group), serum uric acid (mean 5.27 mg/dL vs. 2.95 mg/dL in the control group), and serum phosphorus (mean 5.9 mg/dL vs. 3.74 mg/dL in the control group). There is a statistically non-significant difference between groups regarding serum creatinine and eGFR (Table 2).

**Table 2:** Comparison of laboratory data between the study groups

	Group		Test	
	NS group (*n* = 40)	Control group (n = 40)	*t*	*p*
	Mean ± *SD*	Mean ± *SD*		
Total protein (g/dL)	4.08 ± 0.52	6.63 ± 0.62	− 19.865	< 0.001*
Albumin (g/dL)	2.35 ± 0.61	3.72 ± 0.45	− 11.489	< 0.001*
Prealbumin (mg/dL)	9.18 ± 1.96	18.39 ± 5.7	− 9.667	< 0.001*
Cholesterol (mg/dL)	385.58 ± 113.25	140.25 ± 24.01	13.403	< 0.001*
Uric acid (mg/dL)	5.27 ± 1.25	2.95 ± 0.94	9.362	< 0.001*
Phosphorus (mg/dL)	5.9 ± 1.88	3.74 ± 0.72	6.709	< 0.001*
eGFR^§^ (mL/min/1.73 m^2^)	85.37 ± 14.09	90.74 ± 17.27	− 1.523	0.132
Creatinine^§^ (mg/dL)	0.64 (0.5–2.21)	0.62 (0.4–1.0)	− 0.68	0.497

*t*, independent sample *t*-test; **§**, non-parametric data expressed as median and range and compared using Mann–Whitney test.

*p < 0.05 is statistically significant.

### Ultrasonographic measurements of muscle thickness

On US evaluation of rectus and vastus muscle thickness, both were significantly lower among the NS group. The mean of QRFT was 1.088 cm and 1.27 cm in NS and the control group, respectively, *p* = 0.01, and the mean of QVIT was 0.999 cm and 1.138 cm in NS and the control group, respectively, *p* = 0.017. Regarding subcutaneous fat thickness, it was significantly higher among the NS group (0.745 cm) vs. the control group (0.559 cm), *p* < 0.001 (Table 3).

**Table 3:** Comparison of US measurements between the study groups

	Group		Test	
	NS group (*n* = 40)	Control group (*n* = 40)	*t*	*p*
	Mean ± *SD*	Mean ± *SD*		
QRFT (cm)	1.088 ± 0.355	1.27 ± 0.253	− 2.645	0.01*
QVIT (cm)	0.999 ± 0.333	1.138 ± 0.232	− 2.155	0.017*
SC fat (cm)	0.745 ± 0.275	0.559 ± 0.156	3.172	< 0.001*

*t*, independent sample *t*-test; *NS*, nephrotic syndrome; *QRFT*, quadriceps rectus femoris thickness; *QVIT*, quadriceps vastus intermedius thickness; *SC fat*, subcutaneous fat

*p < 0.05 is statistically significant

### Nutritional intake and muscle wasting

The percent of daily required energy and protein for the study participants and that actually received by them using food diary records were compared between the two groups. It revealed that the NS group received a significantly higher percent of energy (mean 105.23% vs. 91.55% control) and the median percent contribution to total energy intake from protein was lower in the NS group at 9.25% vs. 20% control, and only 37.5% of patients reached the recommended protein intake according to AMDR vs. 72.5% within the control group, *p* = 0.002 (Table 4).

**Table 4:** Comparison between the studied groups and energy and protein intake

	NS group (*n* = 40)	Control group (*n* = 40)	Test (*t*/*Z*)	*p*
	Mean ± *SD*	Mean ± SD		
Energy intake (% recommended)	105.23 ± 20.11	91.55 ± 9.34	3.901	< 0.001*
Protein (% En)^§^	9.25 (4–29%)	20 (6–39%)	− 2.989	0.003*
Protein intake adequacy^+^: Inadequate Adequate	*N* (%) 25 (62.5%) 15 (37.5%)	*N* (%) 11 (27.5%) 29 (72.5%)	9.899^¥^	0.002*

*t*, independent sample *t*-test; *NS*, nephritic syndrome; **§**, non-parametric data expressed as median and range and compared using Mann–Whitney test (*Z*); ¥, chi-square test; %, recommended calculated as the percent ratio of actual intake to recommended intake per child. + Adopted from the acceptable macronutrient distribution range (AMDR). *p < 0.05 is statistically significant

### Correlation study

The QRFT was significantly positively correlated to serum albumin, total protein, and percent of protein intake, while it was significantly negatively correlated with serum uric acid. Also, QVIT was significantly positively correlated to serum albumin and percent of protein intake, while it was significantly negatively correlated with serum uric acid (Table 5).

**Table 5:** Correlation between US measurements of quadriceps muscle thickness and the studied parameters

Parameter	QRFT		QVIT	
	*r*	*p*	*r*	*p*
BMI	0.046	0.684	− 0.024	0.832
Total protein	0.284	0.011*	0.211	0.06
Albumin	0.291	0.009*	0.276	0.013*
PA	0.212	0.059	0.179	0.112
Total cholesterol	− 0.209	0.062	− 0.133	0.239
Uric acid	− 0.362	< 0.001*	− 0.279	0.013*
Phosphorus	− 0.148	0.192	− 0.152	0.178
Disease duration	− 0.237	0.142	− 0.19	0.239
% protein intake	0.755^§^	< 0.001*	0.602^§^	< 0.001*
eGFR	0.037	0.746	− 0.015	0.89
Creatinine	− 0.06§	0.599	0.085§	0.453

*QRFT*, quadriceps rectus femoris muscle thickness; *QVIT*, quadriceps vastus intermedius thickness; *BMI*, body mass index; *r*, Pearson correlation coefficient; §, Spearman rank correlation coefficient; *PA*, prealbumin

*p < 0.05 is statistically significant.

### Factors associated with muscle loss

Only percent of protein intake was significantly independently associated with QRFT and QVIT in a linear stepwise regression analysis of factors significantly correlated with them (for rectus: unstandardized = 0.028, *p* < 0.001; for vastus: unstandardized = 0.02, *p* < 0.001) (Table 6).

**Table 6:** Linear stepwise regression analysis of factors associated with vastus and rectus among the studied participants

		Unstandardized coefficients		Standardized coefficient	*t*	*p*	95% CI	
		*β*	St. error	*Β*			Lower	Upper
QRFT	Constant	0.729	0.05		14.531	< 0.001*	0.629	0.828
	% protein intake	0.028	0.003	0.755	10.178	< 0.001*	0.022	0.033
QVIT	Constant	0.739	0.056		13.183	< 0.001*	0.627	0.851
	% protein intake	0.02	0.003	0.602	6.664	< 0.001*	0.014	0.026

*QRFT*, quadriceps rectus femoris muscle thickness; *QVIT*, quadriceps vastus intermedius thickness; *CI*, confidence interval.

### *Receiver operating characteristic(ROC) curve*

**Fig. 1:**
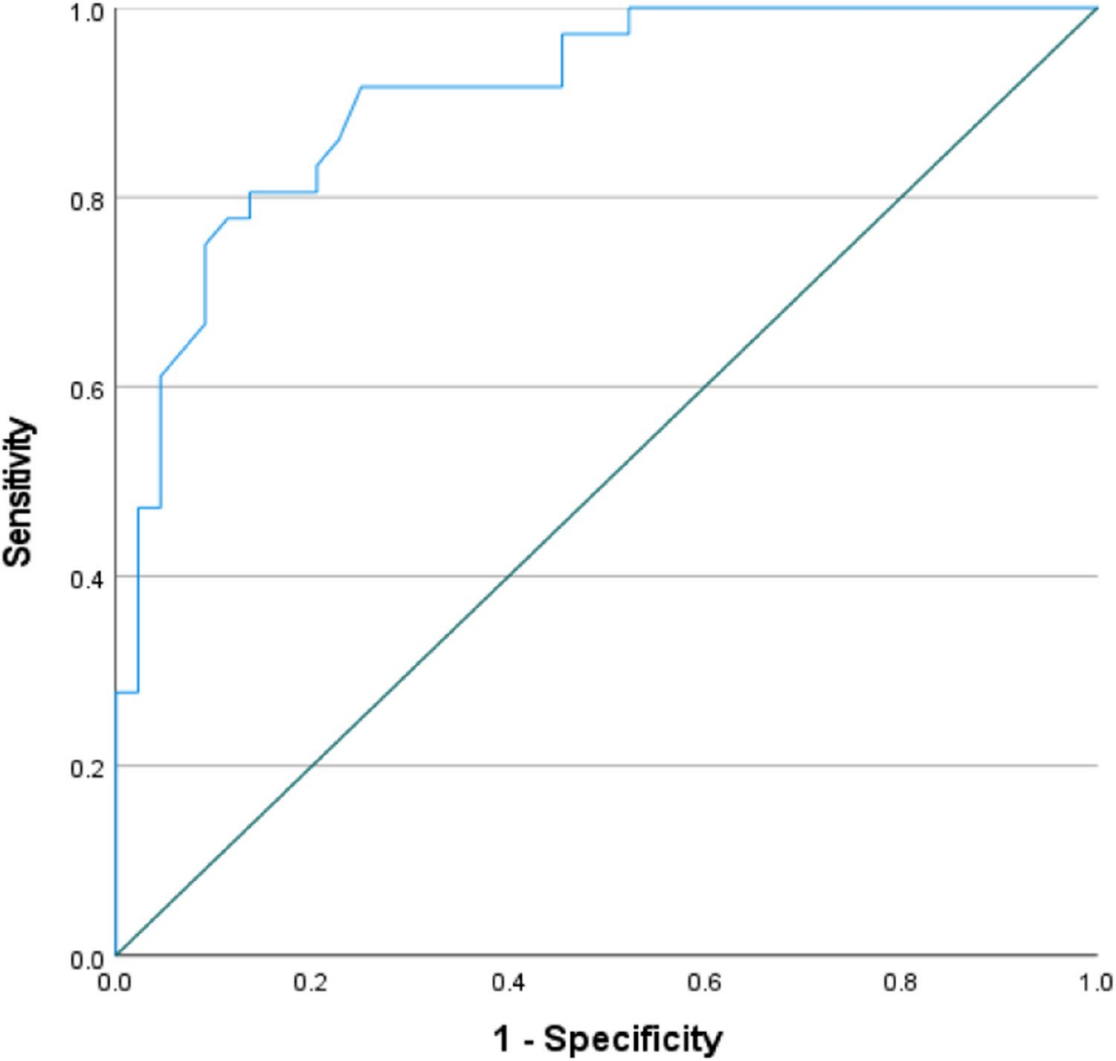
ROC curve showing performance of rectus in prediction of patient at risk of malnutrition

The ROC curve revealed that the best cutoff of QRFT for the prediction of patients at risk of malnutrition was ≤ 1.195 with AUC 0.907, sensitivity 86.1%, specificity 77.3%, positive predictive value 75.6%, negative predictive value 87.2%, and overall accuracy of 81.3% with *p* < 0.001(Fig. 1).

## Discussion

Nutritional status is a crucial determining factor of growth in children with NS, and it must be assessed. There is a higher risk of poor nutritional status in children with NS than in children with other kidney diseases [28].

PEW increases the risk of morbidity and mortality in patients with CKD [29, 30]. The inflammation associated with PEW is a cardiovascular risk factor [12]. PEW in NS, like PEW in CKD, is related to an acceleration in atherosclerosis development, even in children [31]. Patients with severe NS hyperphosphatemia, hyperuricemia, and fluid overload have recognized independent risk factors of cardiovascular events as well [6]. Moreover, PEW causes the patient’s immune system to deteriorate and respond abnormally; as a result, the patient is more vulnerable to infections and their sequelae [32].

ISRNM criteria for PEW are inapplicable to NS, where the requirement of low serum albumin is achieved, yet BMI declines and weight loss is almost never seen, whereas weight increase owing to overhydration is common. Moreover, BMI does not allow for distinction between muscle mass and fat mass. Fluid retention may conceal changes in muscle mass or confound overall weight changes. As a result of these limitations, the loss of muscle mass may be underestimated, causing a failure to precisely distinguish patients who are malnourished or at risk of losing muscle mass and becoming malnourished. Therefore, we have outlined muscle US as a simple bedside method to help with nutritional assessment in this group.

Over the last decade, various methods have been utilized to assess the nutritional status in patients with CKD. However, there is a paucity of data on nutritional status in children with NS, and data concerning US use in NS is limited. To our knowledge, this has been the first clinical study to use this method to permit the early diagnosis of patients with low muscle mass and at risk for the progression to PEW, using a simple tool that can be provided at the patient’s bedside.

In the present study, 40 patients with NS and 40 controls were recruited. BMI was significantly higher in nephrotic children as compared to the healthy control group in agreement with Raikwar et al. and Kuźma-Mroczkowska et al. [33, 34] who concluded that long term (at least 6 months) glucocorticoid therapy is accountable for higher BMI in patients with NS.

In our study, patients with NS had features of hypercatabolism (higher Pi and UA) when compared to the control group. This agrees with a previous study where the NS group presented with elevated phosphorus (*p* = 0.029), uric acid (*p* = 0.002), and blood urea (*p* = 0.049) in comparison with the controls [6]. Hyperphosphatemia and hyperuricemia are well-known hypercatabolism indicators. Feinstein et al. proposed that urinary insulin growth factor 1 excretion and its impacts on higher tubular phosphate retention could be a potential mechanism of phosphorus level elevation in such children [35]. However, as seen in rhabdomyolysis and tumor lysis syndrome, increased cellular mass catabolism could be a further source of serum phosphate [36]. Medications like steroids and diuretics may have a role in uric acid metabolism [10, 37]. Hyperuricemia in children with primary NS was reported as a risk factor for progression to kidney failure [38, 39]. However, in the current study, there is a statistically non-significant difference between groups regarding serum creatinine or eGFR.

Our study revealed that US measurements of rectus and vastus muscle thickness were significantly lower in children with NS than in the control group. This agrees with previous studies that found patients with NS had a significantly lower LBM than the control group by using the BIS [6, 33].

In our case, interstitial edema did not affect muscle size as there was no characteristic ultrasound appearance of hypoechoic muscle edema (which would increase muscle bulk and thickness in its presence) noticed. Only some cases showed subcutaneous edema evident by linear hypoechoic streaks inside echogenic subcutaneous fat. This supports our results regarding significantly lower QRFT and QVIT (absent muscle edema) and higher SC fat thickness (due to SC edema) in the NS group when compared to the control group. This also agrees with another reliability study on quadriceps muscle thickness values measured by US before and after KRT, which found no difference, indicating that even in chronically hyperhydrated patients, US is unaffected by fluid overload and quick and relevant fluid changes [22, 23].

In our study, the NS group received significantly higher percentage of energy and lower percentage of protein, which may be attributed to the consumption of cheap sources of energy like bread in the presence of the extra burden of medication and medical costs in our developing country. Poverty and multiple acute or chronic comorbidities may also play a role in suboptimal nutrient intake [40]. Moreover, one result of the Chronic Kidney Disease in Children (CKiD) study showed that CKD children eat a lot of “empty calorie” foods like fast food, chips (crisps), and other snack foods. Even though they consumed proper macronutrient ranges, their food choices providing the macronutrients were poor on average [41, 42]. In adult studies, a low-protein diet has been shown to help reduce proteinuria [19, 43]. However, in order to ensure proper growth and development in most children and to prevent or treat PEW, increased protein intake closer to 0.8 g/kg/d may be required [44, 45]. Therefore, a balance should exist between cardiovascular/psychological outcomes, risks of being overweight and obesity, and appropriate energy and protein intake [46].

In our study, muscle thickness was significantly positively correlated with serum albumin and percent of protein intake, while it was significantly negatively correlated with serum uric acid, in agreement with Matyjek et al. [6] who found a strong negative correlation between lean tissue mass and blood urea concentration. These findings support the theory that those patients had augmented muscle protein degradation. Moreover, a prior study compared parenteral nutrition with an amino acid intake of 0.8 g/kg/day or 1.2 g/kg/day among 119 critically ill patients and concluded that higher amino acid intake was linked to a significant increase in forearm muscle thickness as measured by ultrasound [47]. On the other hand, another observational study of 29 critically ill patients did not find a correlation between muscle loss and either caloric or protein debt over the first week [48].

According to our findings, the percentage of protein consumed was significantly associated with quadriceps muscle thickness as measured by US. The pathogenesis of muscle wasting in CKD is multifactorial. Inadequate nutrient intake, which is quite common in these patients, is recognized as the most important factor contributing to the development of muscle wasting [18].

The best cutoff of quadriceps muscle thickness as determined by the US in the prediction of patients at risk of malnutrition is 1.195 with an area under curve of 0.907, sensitivity of 86.1%, specificity of 77.3%, positive predictive value of 75.6%, negative predictive value of 87.2%, and overall accuracy of 81.3% with *p* < 0.001. Hence, muscular US are reliable, practical, and accessible tools beneficial for the estimation of muscle mass and for the follow-up of the nutritional status of patients with NS.

## Strength and limitations

The current study has a number of strengths. The main strength is that this is the first study to use muscle US in children with NS for assessment of nutrition status. The findings of our study should serve as a reminder of the importance of routine nutritional assessment in children with NS for early detection, treatment, and nutritional rehabilitation counseling, all of which can contribute to a reduction in mortality. The present study also has some limitations. The sample size and the single-center sample are two limitations. However, the results of this study are quite interesting due to the extremely accurate information offered by ultrasonography. Future research must replicate this method with bigger sample sizes to verify lower limb muscle thickness in nutritional status assessment and to provide reference standards based on age and gender in children with NS. Prospective evaluation of US assessing changes in muscle status as a focus on the outcome of nutritional intervention in PEW treatment is highly recommended. On the other hand, other limitations of the present study are the unmeasured confounding effects that may still exist despite adjusting for potential confounders by multiple regressions and the absence of conclusive evidence from other markers of malnutrition. The validity of the ultrasonic assessment for determining nutritional profiles might be improved by more studies using confirmative samples of malnutrition in children with NS.

## Conclusion

In children with NS, quadriceps femoris US could be a simple, precise, and non-invasive means of assessment of muscle wasting in patients with PEW. Consequently, utilizing a US technique in a clinical context should be taken into consideration for a quick screening of PEW risk.

## Supplementary Information

Below is the link to the electronic supplementary material.

Graphical Abstract (PPTX 52 KB)

## Data Availability

The datasets created and/or analyzed during the current work are accessible upon reasonable request from the corresponding author.
