# Supplementary material for: Utility of muscle ultrasound in nutritional assessment of children with nephrotic syndrome
Source: Pediatr Nephrol. 2022 Nov 10;38(6):1821–9. doi: 10.1007/s00467-022-05776-y (PMC10154282; doi:10.1007/s00467-022-05776-y)
Supplement: Supplementary file 1 — Graphical Abstract (PPTX 52 KB) [file 467_2022_5776_MOESM1_ESM.pptx]

## Slide 1
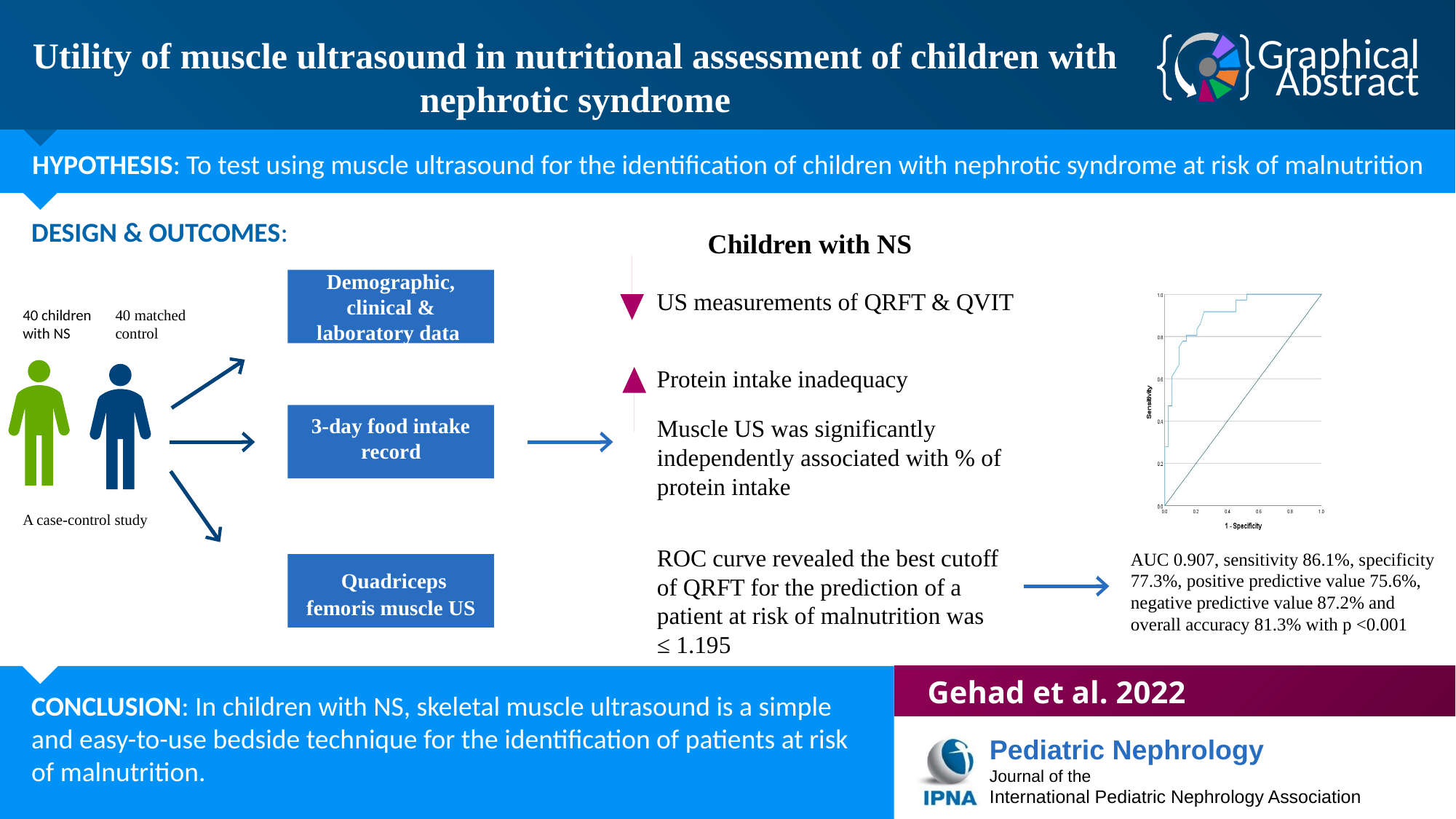

Utility of muscle ultrasound in nutritional assessment of children with nephrotic syndrome
HYPOTHESIS: To test using muscle ultrasound for the identification of children with nephrotic syndrome at risk of malnutrition
DESIGN & OUTCOMES:
Children with NS
Demographic, clinical & laboratory data
US measurements of QRFT & QVIT
40 children with NS
40 matched control
Protein intake inadequacy
3-day food intake record
Muscle US was significantly independently associated with % of protein intake
A case-control study
ROC curve revealed the best cutoff of QRFT for the prediction of a patient at risk of malnutrition was
≤ 1.195
AUC 0.907, sensitivity 86.1%, specificity 77.3%, positive predictive value 75.6%, negative predictive value 87.2% and overall accuracy 81.3% with p <0.001
 Quadriceps femoris muscle US
Gehad et al. 2022
CONCLUSION: In children with NS, skeletal muscle ultrasound is a simple and easy-to-use bedside technique for the identification of patients at risk of malnutrition.
